# Race, Gender, and Faculty Retention in Academic Medicine

**DOI:** 10.1001/jamanetworkopen.2024.45143

**Published:** 2024-11-14

**Authors:** Taneisha S. Scheuermann, Lauren Clark, Nasrin Sultana, Nathalia Machado, Elena Shergina, Deepika Polineni, Grace H. Shih, Robert D. Simari, Jo A. Wick, Kimber P. Richter

**Affiliations:** 1 Department of Population Health, University of Kansas School of Medicine, Kansas City; 2 Department of Biostatistics and Data Science, University of Kansas School of Medicine, Kansas City; 3 Adult and Child Center for Outcomes Research and Delivery Science, University of Colorado School of Medicine, Aurora; 4 Department of Pediatrics, Division of Allergy and Pulmonary Medicine, Washington University School of Medicine, St. Louis, Missouri; 5 Department of Anesthesiology, University of Kansas School of Medicine, Kansas City; 6 Department of Cardiovascular Medicine, University of Kansas School of Medicine, Kansas City

## Abstract

**Importance:**

Poor retention may be associated with lack of faculty diversity in academic medicine.

**Objective:**

To examine differences in faculty retention by gender, degree type, and race and ethnicity using data from US medical schools.

**Design, Setting, and Participants:**

This cohort study analyzed data from 155 medical schools for 1978 to 2021, which were obtained from the Association of American Medical Colleges. Retention rates were calculated by gender, race and ethnicity, and degree type. Time to attrition was estimated using Kaplan-Meier curves and adjusted Cox proportional hazards models were used to examine the differences in retention by gender and race. Data were analyzed from March 2021 to November 2022.

**Main Outcomes and Measures:**

The duration of continuous employment in academic medicine and variations in retention based on gender, race, ethnicity, and terminal degree.

**Results:**

Among 390 766 faculty members (232 829 [59.6%] men; 1190 American Indian, Alaska Native, Native Hawaiian, or Pacific Islander [0.3%]; 72 490 Asian [18.6%]; 14 920 Black [3.8%]; 20 345 Hispanic, Latino, of Spanish origin, or multirace Hispanic [5.2%]; 251 670 non-Hispanic White [64.4%] participants; and 30 151 [7.7%] other race), 259 628 (66.4%) had MD or MD-equivalent terminal degrees. Women left academic medicine a median of 1 year earlier (median retention, 8.33 years; 95% CI, 8.18-8.41 years) than men (median retention, 9.35 years; 95% CI, 9.25-9.42 years). Compared with White faculty (median retention, 9.91 years; 95% CI, 9.83-9.92 years), multiracial and faculty of other races had the lowest retention (median retention, 5.42 years; 95% CI, 5.33-5.55 years), followed by Alaska Native, American Indian, Native Hawaiian, and Pacific Islander (median retention, 6.00 years; 95% CI, 5.62-6.62 years), Black (median retention, 7.78 years; 95% CI, 7.50-8.00 years), Asian (median retention, 8.00 years; 95% CI, 8.00-8.11 years) and Hispanic faculty (median retention, 9.00 years; 95% CI, 8.75-9.16 years). Women faculty in the 1970s were 20% more likely than men to leave academia, whereas women in the 2010s had no greater risk of leaving, mainly due to a decline in retention of men.

**Conclusions and Relevance:**

Gender differences in faculty retention have decreased in the most recent decade; however, men had lower retention compared with earlier cohorts. In this study, faculty who were underrepresented in medicine left academic medicine a median of 1 to 4 years earlier than White faculty. Greater attrition among men and women for recent cohorts suggests that enhanced retention efforts are imperative to maintain an experienced and diverse faculty.

## Key Points

**Question:**

Does faculty retention in academic medicine differ by gender, race and ethnicity, and degree type?

**Findings:**

In this cohort study of 390 766 faculty members from 1978 to 2021, women left academic medicine a median of 1 year earlier than men; however, there was no observed gender difference in the 2010s due to increased attrition among men. Compared with faculty from other racial and ethnic groups, White faculty had the longest median retention of 10 years.

**Meaning:**

These findings suggest that the gender gap in faculty retention closed in the 2010s due to increasing attrition among men; however, racial and ethnic minority faculty still experienced greater attrition compared with White faculty.

## Teaser

This cohort study evaluates differences in faculty retention in US medical schools fro 1978 to to 2021 by gender, degree type, and race and ethnicity.

## Introduction

Enhancing gender, racial, and ethnic diversity in medical school faculty could improve health care clinician training, better meet the needs of medically underserved populations through both clinical practice and research, and reduce disparities in health care quality.^1,2,3^ In 2021, women represented 43% of medical school faculty; by rank, the proportion of women faculty ranged from 59% of instructors to only 28% of full professors.^4^ Although racial and ethnic diversity has increased among medical school faculty over the past few decades, Black, Hispanic, American Indian, Alaska Native, and Pacific Islander individuals, as well as women, remain underrepresented in medicine (UIM) relative to the US population.^5,6^

Poor retention negatively impacts women’s and racial and ethnic minority groups’ representation among medical school faculty.^7,8^ Although medical school faculty are composed of interdisciplinary educators, researchers, and clinicians, prior studies have generally focused on clinical faculty and on medical school graduates, or not reported outcomes by degree type. Medical school MD faculty who are women and from racial and ethnic groups that are UIM populations were at greater risk for attrition at 10-year follow-up using faculty roster data from the Association of American Medical Colleges (AAMC) between 2000 and 2012.^8^ Similarly, a national longitudinal survey study that sampled faculty from 24 medical schools in 1995 showed that UIM individuals were less likely to be retained over 17-year follow-up.^9^ One study examined the association between retention and type of appointment using data from a single medical school.^10^ In that study, women vs men and clinical and auxiliary track faculty vs tenure track faculty were at greater risk of attrition.^10^ Important gaps remain in our understanding of retention as 25% of medical school faculty are not MDs.^4^

In 2009, the Liaison Committee for Medical Education accreditation standards began including policies and procedures for ensuring a diverse student body and faculty.^11^ Several studies focus on programs to reduce attrition, indicating that institutions have been making efforts to address these standards. If medical schools have implemented policies to address faculty diversity that successfully impacted faculty retention, we would expect these rates to improve for more recent cohorts.

This secondary analysis delineates trends in retention for all medical school faculty by gender, race and ethnicity, and terminal degree using national data spanning 43 years. We examine whether retention differs by gender, race and ethnicity, and terminal degree, as well as by decade to determine whether gaps in retention are closing over time.

## Methods

### Data Source and Study Sample

The AAMC faculty roster data for this study includes deidentified information on full-time faculty positions for the academic years 1978 to 1979 through 2019 to 2020. We further restricted the data to only faculty whose first academic position started after January 1, 1978. This analysis of deidentified data did not constitute human participants research in accordance with 45 CFR §46 and did not require institutional review board review or informed consent. Reporting of this study followed the Strengthening the Reporting of Observational Studies in Epidemiology (STROBE) reporting guideline for cohort studies.

### Study Measures

We calculated the length of continuous employment in academic medicine as the time between first appointment starting date and latest appointment ending date, allowing a gap of up to 367 days between appointments. If there was no end date, we assumed continued continuous employment. Faculty with an end date but no new appointment after 367 days were considered to have left academic medicine. Data on faculty roster appointments contained information on degree type, appointment type, department type, starting and end date of the appointments, and degree type. Degree type categories were MD or MD-equivalent, including DO, MBBS, or MBChB; PhD or equivalent; and both MD (or MD-equivalent) and PhD degrees and other degrees (degree other than MD or PhD or their equivalent). Demographic information included gender, birth year, and race or ethnic group of the faculty as reported to the AAMC from institutional faculty records. We categorized participants into Other race if this was indicated as unknown, other, or non-Hispanic multiracial. Institutional characteristics included whether the medical school was public or private, quartile of total revenues for public and private institutions, and institution size (small, medium, or large).

### Statistical Analyses

To describe retention across all cohorts, we calculated the proportion retained for several milestone years (at least 3, 5, 7, 10, 15, and 25 years of retention) for both men and women with the corresponding 95% exact binomial CIs. We graphed proportions retained by milestone to illustrate trends over time for men and women and gender differences by milestone. For each degree type, we also calculated the proportion retained at milestone years to show patterns in faculty retention. We examined retention milestones for the most recent cohort of faculty with appointments beginning in 2010 and calculated the proportion retained by race and ethnicity and gender categories for 3-, 5-, 7-, and 10-year milestones.

For our time-to-event analysis, we used nonparametric Kaplan-Meier curves to estimate median retention and depict attrition from academic medicine by gender, degree type, and race or ethnic group. We restricted follow-up time to 40 years, censoring any observations with follow-up time greater than 40 years. In addition, faculty who were still continuously employed (ie, had no end date) were censored within the analyses. We estimated differences between genders in the average probability of attrition using a Cox proportional hazards (PH) model. This model included gender, degree type, race or ethnic group, and an interaction between gender and degree type. The interaction term allowed us to examine changes in gender outcomes for each degree type. We conducted a sensitivity analysis adjusting for gender, degree, race and ethnicity, and institutional characteristics (public vs private, total revenue quartile for private institutions, total revenue quartile for public institutions, and size of institution).

To examine differences in retention between men and women by decade, we further restricted follow-up time to 10 years so that each decade cohort would have approximately the same length of follow-up. We also excluded people whose first appointment started after January 1, 2020. We used a Cox PH model to estimate the hazard ratios of attrition for women vs men by decade, adjusting for degree type and race. Analyses were conducted using SAS version 9.4 (SAS Institute) and RStudio version 4.1.2 (R Project for Statistical Computing). Data were analyzed from March 2021 to November 2022.

## Results

This study sample consisted of 390 766 faculty members (1190 American Indian, Alaska Native, Native Hawaiian, or Pacific Islander [0.3%]; 72 490 Asian [18.6%]; 14 920 Black [3.8%]; 20 345 Hispanic, Latino, of Spanish origin, or multirace Hispanic [5.2%]; 251 670 non-Hispanic White [64.4%] participants; and 30 151 [7.7%] other race) from 155 US medical schools (Table 1). The majority of faculty members were men (232 829 participants [59.6%]) and the median (IQR) age of first appointment was 35 (32-40) years. The majority of medical school faculty held MD or MD-equivalent terminal degrees (259 628 participants [66.4%]) and 87 954 (22.5%) held PhDs. A slightly greater proportion of men compared with women earned both an MD/MD-equivalent and PhD (or PhD equivalent) (18 269 [7.8%] vs 7184 participants [4.5%], respectively) and more women earned other degrees compared with men (13 135 [8.3%] vs 4596 participants [2.0%], respectively). Faculty were almost evenly distributed between public (174 852 participants [51.1%]) and private institutions (167 595 participants [48.9%]).

**Table 1.** Demographic Information for All Faculty

Variable	Participants, No. (%)		
	Men (n = 232 829)	Women (n = 157 937)	All (N = 390 766)
Race and ethnicity			
American Indian, Alaska Native, Native Hawaiian, or Pacific Islander	682 (0.3)	508 (0.3)	1190 (0.3)
Asian	42 687 (18.3)	29 803 (18.9)	72 490 (18.6)
Black	6644 (2.9)	8276 (5.2)	14 920 (3.8)
Hispanic, Latino, of Spanish origin, or multirace Hispanic	11 784 (5.1)	8561 (5.4)	20 345 (5.2)
White	154 463 (66.3)	97 207 (61.5)	251 670 (64.4)
Other^a^	16 569 (7.1)	13 582 (8.6)	30 151 (7.7)
Degree type			
MD only	159 503 (68.5)	100 125 (63.4)	259 628 (66.4)
MD and PhD	18 269 (7.8)	7184 (4.5)	25 453 (6.5)
PhD only	50 461 (21.7)	37 493 (23.7)	87 954 (22.5)
Other degree	4596 (2.0)	13 135 (8.3)	17 731 (4.5)
Approximate age at first appointment, y			
Mean (SD)	37.9 (8.10)	36.7 (7.16)	37.40 (7.76)
Median (IQR)	35.0 (32.0-41.0)	35.0 (32.0-40.0)	35.0 (32.0-40.0)
Institutional variables			
No.	201 024	141 423	342 447
Institution type			
Public	104 382 (51.9)	70 470 (49.8)	17 4852 (51.1)
Private	96 642 (48.1)	70 953 (50.2)	167 595 (48.9)
Public institution funding variable, quartile			
1	7587 (3.8)	4791 (3.4)	12 378 (3.6)
2	18 279 (9.1)	11 219 (7.9)	29 498 (38.6)
3	30 582 (15.2)	18 780 (13.3)	49 362 (14.4)
4	47 934 (23.8)	35 680 (25.2)	83 614 (24.4)
Private institution funding variable, quartile			
1	4495 (2.2)	2588 (1.8)	7083 (2.1)
2	15 391 (7.7)	11 951 (8.5)	27 342 (8.0)
3	29 015 (14.4)	21 916 (15.5)	50 931 (14.9)
4	47 741 (23.7)	34 498 (24.4)	82 239 (24.0)
Institution size			
Small	27 063 (13.5)	17 195 (12.2)	44 258 (12.9)
Medium	76 421 (38.0)	54 407 (38.5)	130 828 (38.2)
Large	97 540 (48.5)	69 821 (49.4)	167 361 (48.9)

Abbreviation: HR, hazard ratio.

^a^ Other includes unknown, other, or non-Hispanic multiracial.

### Unadjusted Analyses

#### Retention Across Careers

Faculty were employed in academic medicine for a median of 8.97 (95% CI, 8.92-9.00) years. The majority of both men and women were employed at 1 institution during their career in academic medicine (143 2249 women [90.7%] and 203 027 men [87.2%]), and 12 793 women (8.1%) and 24 680 men (10.6%) were employed at 2 institutions. Throughout their careers, most faculty were in clinical departments (139 300 women [88.2%] and 201 164 men [86.4%]), followed by basic science (13 267 women [8.4%] and 24 447 men [10.5%]), and other department types (2053 women [1.3%] and 1630 men [0.7%]); 3317 women (2.1%) and 5821 men (2.5%) changed department types.

**Figure 1.**
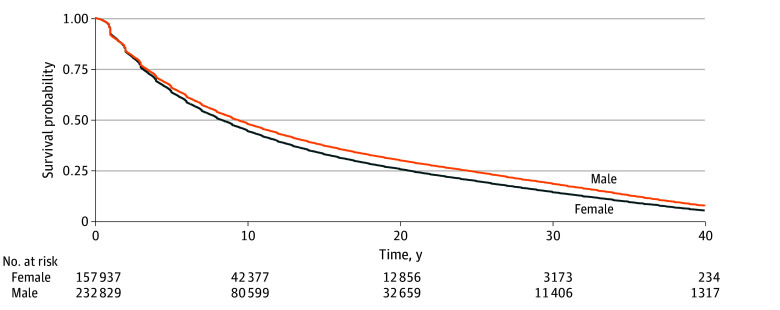
Retention Following Initial Appointment to Medical Faculty by Gender (N = 390 766) Figure 1 depicts the Kaplan-Meier survival curve for probability of being retained in academic medicine after first appointment to any rank (instructor, assistant professor, associate professor, or full professor). Median (IQR) retention for women was 8.33 (8.18-8.41) years and for men, 9.35 (9.25-9.42) years.

**Figure 2.**
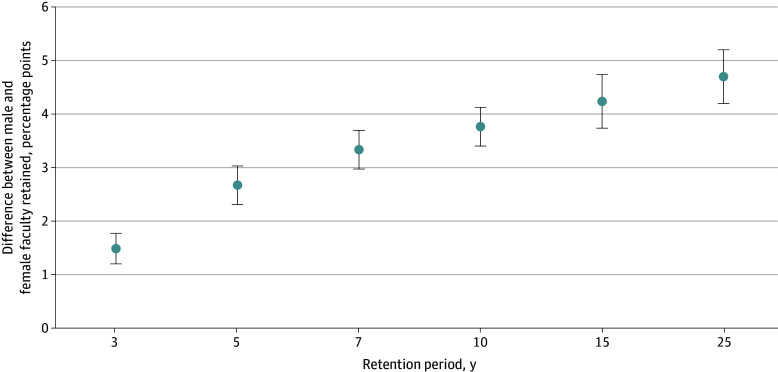
Percentage Point Differences Between Male and Female Faculty Retained by Career Milestones Whiskers indicate 95% CIs.

Across all cohorts, women were retained in academic medicine for a median of 8.33 (95% CI, 8.18-8.41) years compared with a median of 9.35 (95% CI, 9.25-9.42) years for men. Figure 1 shows that this gap in retention persisted over time. Women were retained at lower proportions compared with men at every important milestone (3 years, 5 years, and so forth). We observed an increase in the retention gap for women vs men over the length of careers with a 1.5 percentage point lower retention between women and men at 3 years and 4.7 percentage point lower retention at 25 years (Figure 2). At 3 years after first appointment, 107 494 women (75.9%) were retained compared with 167 716 men (77.4%); at 10 years, 42 377 women (45.1%) were retained compared with 80 599 men (48.9%); and at 25 years, 7085 women (21.8%) were retained compared with 20 868 men (26.5%) (eFigures 1-2 and eTables 1-5 in Supplement 1 show retention milestones by gender across degrees).

By degree type, joint MD/PhD recipients had the longest median retention at 12.67 (95% CI, 12.33-13.00) years, followed by PhDs at 9.67 (95% CI, 9.50-9.83) years, then MDs and MD-equivalent degrees at 8.62 (95% CI, 8.50-8.75) years (see eTable 6 and eFigures 3-4 in Supplement 1). The median retention time for faculty with other degrees was only 6.54 (95% CI, 6.37-6.75) years. Retention across race and ethnicity ranged from a median time of 9.91 (95% CI, 9.83-9.92) years for non-Hispanic White faculty to a low of 5.42 (95% CI, 5.33-5.55) years for faculty in the Other race category. Other faculty retention was as follows: American Indian, Alaska Native, Native Hawaiian, or Pacific Islander (median retention, 6.00 years; 95% CI, 5.62-6.62 years), Black (median retention, 7.78 years; 95% CI, 7.50-8.00 years), Asian (median retention, 8.00 years; 95% CI, 8.00-8.11 years), and Hispanic faculty (median retention, 9.00 years; 95% CI, 8.75-9.16 years).

#### Retention by Race, Ethnicity, and Gender

Among the cohort with appointments beginning in 2010s, larger differences in retention rates appear across race and ethnicity compared with gender differences within each racial and ethnic group for each of the 4 milestones examined (3, 5, 7, and 10 years) (see eTable 7 and eFigure 5 in Supplement 1). Non-Hispanic White men had the highest retention rate at each milestone (78.2%, 66.9%, 57.5%, and 48.6% at 3-, 5-, 7-, and 10-year milestones, respectively). Men who reported Other race had the lowest retention (63.0%) at the 3-year milestone, and American Indian, Alaska Native, Native Hawaiian, or Pacific Islander men had the lowest retention at each subsequent milestone (41.9%, 29.4%, and 13.9% at 5-, 7-, and 10-year milestones, respectively). At 3 years after first appointment, Asian, Hispanic, and non-Hispanic White women had a 1 to 2 percentage point lower retention compared with men of the same race and ethnicity, whereas Black women and women reporting Other race had a 1 percentage point higher retention relative to men of the same race and ethnicity. The largest gender difference by race and ethnicity was an 8-percentage point difference observed between American Indian, Alaska Native, Native Hawaiian, or Pacific Islander women (72.8%) and men (64.9%) retained at 3 years. The pattern of gender differences at 5 and 7 years after first appointment was similar to the 3-year milestone, except that there was no gender difference for faculty reporting Other race at 5- and 7-year milestones and for Asian and Black faculty at the 7-year milestone. Ten years after first appointment, less than half of faculty were retained; White men (48.6%), Asian men (43.3%), and Black men (38.7%) had higher retention rates compared with White women (44.6%), Asian women (40.1%), and Black women (38.4%). Among American Indian, Alaska Native, Native Hawaiian, or Pacific Islander and Hispanic faculty, women had greater retention compared with men (33.3% for American Indian, Alaska Native, Native Hawaiian, or Pacific Islander women vs 13.9% for American Indian, Alaska Native, Native Hawaiian, or Pacific Islander men; 44.7% for Hispanic women vs 43.5% for Hispanic men). There was no gender difference for faculty reporting Other race.

### Adjusted Cox Proportional Hazards Models

In an adjusted Cox PH model for all cohorts including gender, degree, and race and ethnicity, women were at greater risk of attrition among PhD, MD and MD-equivalent, and joint PhD and MD holders, as well as within each racial and ethnic group (Table 2). Compared with men, women faculty with PhDs were 11% more likely to leave academic medicine (hazard ratio [HR], 1.11; 95% CI, 1.09-1.13). Somewhat smaller gaps were observed for women with MDs (HR, 1.06; 95% CI, 1.05-1.08), and joint MD and PhD degrees compared with men (HR, 1.04; 95% CI, 1.00-1.08). Retention was similar between women and men faculty holding other degrees (HR, 1.01; 95% CI, 0.97-1.05). Racial and ethnic minority faculty had the highest risks for attrition. Compared with non-Hispanic White faculty, faculty identifying as other or multiracial (HR, 1.57; 95% CI, 1.54-1.59) and American Indian, Alaska Native and Native Hawaiian, or Pacific Islander (HR, 1.52; 95% CI, 1.42-1.62) were at over 50% greater risk for attrition, Black and Asian faculty had 17% greater risk of attrition, and Latinx faculty had 6% greater risk. When institutional variables including private vs public, total revenue quartile, and institutional size were added to the adjusted Cox PH model, the overall pattern of associations did not change with 1 exception: retention between women and men faculty holding joint MD and PhD degrees was similar (HR, 0.99; 95% CI, 0.96-1.04) (see eTable 8 in Supplement 1).

**Table 2.** Cox Proportional Hazard Model Results With Interaction Between Gender and Degree Type, Including Race

Variable	Leaving academia (No. of events)	Median retention (95% CI)	HR (95% CI)
Gender			
MD only			
Women (n = 100 125)	57 707	8.08 (8.00-8.21)	1.07 (1.05-1.08)
Men (n = 159 503)	101 033	8.92 (8.83-9.00)	1 [Reference]
MD and PhD			
Women (n = 7184)	3744	12.00 (11.58-12.75)	1.04 (1.00-1.08)
Men (n = 18 269)	10 636	12.92 (12.50-13.25)	1 [Reference]
PhD only			
Women (n = 37 493)	22 612	9.00 (8.91-9.16)	1.11 (1.09-1.13)
Men (n = 50 461)	32 392	10.17 (10.00-10.34)	1 [Reference]
Other degree			
Women (n = 13 135)	8841	6.42 (6.25-.6.61)	1.01 (0.97-1.05)
Men (n = 4596)	3316	6.91 (6.58-7.12)	1 [Reference]
Race and ethnicity			
American Indian, Alaska Native, Native Hawaiian, or Pacific Islander (n = 1190)	822	6.00 (5.62-6.62)	1.52 (1.42-1.62)
Asian (n = 72 490)	41 040	8.00 (8.00-8.11)	1.18 (1.16-1.19)
Black (n = 14 920)	9294	7.78 (7.50-8.00)	1.17 (1.15-1.19)
Hispanic, Latino, of Spanish origin, or multirace Hispanic (n = 20 345)	11 432	9.00 (8.75-9.16)	1.06 (1.04-1.08)
White (n = 251 670)	157 408	9.91 (9.83-9.92)	1 [Reference]
Other (n = 30 151)^a^	20 285	5.42 (5.33-5.55)	1.57 (1.54-1.59)

Abbreviation: HR, hazard ratio.

^a^ Other includes unknown, other, or non-Hispanic multiracial.

Finally, we examined retention by gender and decade of first appointment from the 1970s to the 2010s, adjusted for degree type and race and ethnicity and restricting follow-up time to 10 years (Table 3). In these analyses, we found that women who joined the faculty in the 1970s were 20% more likely to leave academia, whereas women who joined the faculty in the 2010s had no greater risk of leaving compared with men (Table 3). Of note, the median retention time for women in the 1970s was 7.17 (95% CI, 6.75-8.00) years and in the 2010s the median was 7.83 (95% CI, 7.74-7.92) years. The gap decreased due to a decline in men’s tenure in academic medicine, with men in the 1970s retained for a median greater than 10 years and those joining the faculty in the 2010s being retained for a median of 8.08 (95% CI, 8.00-8.25) years. After controlling for the outcomes of private vs public institution, total revenue, and institution size, the pattern of gender differences remained the same (see eTable 9 in Supplement 1).

**Table 3.** Cox Proportional Hazard Model Results With Interaction Between Gender and Decade (10 Years’ Follow-Up), Including Degree Type and Race^a^

Variable	Leaving academia (No. of events)	Median retention (95% CI)	HR (95% CI)
Degree type			
MD only (n = 258 111)	122 779	8.60 (8.50-8.74)	1 [Reference]
MD and PhD (n = 25 371)	10 228	DNE	0.73 (0.71-0.74)
PhD only (n = 87 147)	39 735	9.67 (9.50-9.83)	0.85 (0.84-0.86)
Other degree (n = 17 456)	9553	6.50 (6.34-6.72)	1.16 (1.13-1.18)
Gender			
1970s			
Women (n = 2255)	1297	7.17 (6.75-8.00)	1.20 (1.13-1.28)
Men (n = 9434)	4680	DNE (9.83-DNE)	1 [Reference]
1980s			
Women (n = 15 009)	8237	8.41 (8.00-8.66)	1.07 (1.05-1.10)
Men (n = 39 608)	20 326	9.50 (9.33-9.75)	1 [Reference]
1990s			
Women (n = 25 587)	13 477	9.25 (9.00-9.42)	1.05 (1.03-1.07)
Men (n = 46 789)	23 445	10.00 (9.92-DNE)	1 [Reference]
2000s			
Women (n = 44 496)	24 748	8.17 (8.04-8.35)	1.09 (1.07-1.11)
Men (n = 60 982)	31 537	9.50 (9.33-9.62)	1 [Reference}
2010s			
Women (n = 69 219)	26 249	7.83 (7.74-7.92)	1.01 (0.99-1.03)
Men (n = 74 706)	28 299	8.08 (8.00-8.25)	1 [Reference]
Race and ethnicity			
American Indian, Alaska Native, Native Hawaiian, or Pacific Islander (n = 1183)	694	6.00 (5.62-6.62)	1.49 (1.39-1.61)
Asian (n = 71 840)	34 131	8.00 (8.00-8.10)	1.18 (1.17-1.19)
Black (n = 14 804)	7331	7.77 (7.50-8.00)	1.16 (1.13-1.19)
Hispanic, Latino, of Spanish origin, or multirace Hispanic (n = 20 168)	9144	9.00 (8.75-9.14)	1.05 (1.03-1.08)
White (n = 250 265)	113 721	9.91 (9.83-9.92)	1 [Reference]
Other (n = 29 825)^b^	17 274	5.42 (5.33-5.53)	1.61 (1.58-1.63)

Abbreviations: DNE, does not exist; HR, hazard ratio.

^a^ Observations that started in 2020s or later were excluded as follow-up was less than 2 years, resulting in 388 085 observations. Because follow-up time was restricted to 10 years, medians are not displayed for groups with median retention longer than 10 years.

^b^ Other includes unknown, other, or non-Hispanic multiracial.

## Discussion

This analysis of 4 decades of faculty data from 155 US medical schools suggests over the course of their careers, women and UIM faculty experience markedly worse retention compared with male and White faculty. UIM faculty leave academic medicine a median of 1 to 4 years earlier than White faculty. For women, the retention gap with men grows larger with longer milestones ranging from 3-year to 25-year retention. Notably, women starting careers in academic medicine in the 2010s have similar retention to men of the same epoch. Because this is mainly due to a decrease in faculty retention among men, this suggests that attrition is getting worse for all. While the gender gap has narrowed, UIM faculty still experience greater attrition compared with their non-Hispanic White peers in the most recent cohort becoming faculty in the 2010s.

As we grapple with significant racial and ethnic health disparities in the US, recruiting and retaining a diverse faculty in academic medicine is critical to improving population health outcomes and enhancing health equity.^1,2,3^ Therefore, high rates of attrition observed among UIM faculty are cause for concern. American Indian and Alaska Native individuals have the lowest life expectancy in the US,^12^ are UIM populations,^5^ and have the shortest careers in academic medicine, with 50% greater attrition than White faculty. Compared with White faculty, Black and Hispanic faculty are also at greater risk for attrition. Although not all Asian ethnic groups are UIM populations, it is important to note that the rate of attrition is also higher than for White faculty.

Our findings mirror those of prior studies examining differences in faculty retention by race, ethnicity, and gender and extend our understanding of retention differences by training background. Similar to earlier studies,^7,8,9,10,11^ we find that women and UIM faculty are retained at lower rates than men and White faculty; but also show a narrowing gender gap in the 2010s. Extending other studies, we find that retention differed by degree type. We found the largest retention gap between women and men with PhDs with women being at 11% greater risk of attrition. Both women MDs and MD-PhDs experienced greater risk of attrition relative to men (7% and 4%, respectively).

Faculty leave academic medicine for a variety of reasons, including unsupportive institutional culture, dissatisfaction with career advancement, lack of mentors or opportunities for faculty development, frustrations with research, and salary.^13,14,15,16^ Particularly for clinical faculty, debt and higher pay in nonacademic settings could drive attrition. These factors may also drive down diversity in academic medicine. Medical schools that provide faculty development programs and other supports for faculty experience increases in retention.^17,18^ In addition to faculty development programs, strategies recommended for improving retention of a diverse faculty include facilitating culture change, ensuring equitable distribution of clinical and research commitments, and promoting and expanding loan repayment programs.^19^

### Strengths and Limitations

Study strengths include its large sample size, inclusion of degree type, and use of survival analysis and adjusted hazards regression. The racial and ethnic distribution of women faculty could have accounted for retention differences; however, adjusted models found that it did not. A major limitation of this study is that we did not adjust for productivity or appointment type. We also excluded those who left and then returned to academic medicine after more than a year. Further, the dataset analyzed in this study only includes full-time appointments; as a result, full-time faculty who became part-time or volunteer faculty are treated as having left academic medicine. Therefore, the findings of this study may not generalize to faculty employed part-time in academic medicine. Finally, our data do not describe the roles that faculty leaving academic medicine pursue, which may include other academic or clinical appointments.

## Conclusions

There are significant societal and institutional impacts when faculty leave academic medicine early in their careers. The education and professional training needed to achieve midcareer status involves significant amounts of investment—much of this is lost when more than half of faculty leave within 8 years.^20^ Diversifying faculty should boost diversity in the clinical workforce. Women and ethnic and racial groups with low representation, however, have the shortest careers in academic medicine. Academic medicine must develop strategies to recruit and retain a strong workforce to both improve equity^21^ and ensure meaningful career trajectories for the entire faculty body.

## Notes

Supplement 1. **eFigure 1.** Proportion Retained by Career Milestones **eFigure 2.** Survival Curve for Gender by Degree Type **eFigure 3.** Survival Curve for Degree Type **eFigure 4.** Survival Curve for Race and Ethnicity **eFigure 5.** Proportion Retained by Gender and Race/Ethnicity by Career Milestones **eTable 1.** Proportion Retained by Career Milestones **eTable 2.** Proportion of MD Only Faculty Retained by Career Milestones **eTable 3.** Proportion of PhD Only Faculty Retained by Career Milestones **eTable 4.** Proportion of Joint MD and PhD Faculty Retained by Career Milestones **eTable 5. **Proportion of Other Degree Faculty Retained by Career Milestones **eTable 6. **Bivariable Hazard Ratios for Attrition by Gender, Race, Degree Type, and Decade **eTable 7.** Proportion of Faculty Retained by Race and Ethnicity and Gender **eTable 8.** Cox PH Model Results With Interaction Between Gender and Degree Type, Including Race and Institutional Variables

Supplement 2.
Data Sharing Statement
