# Supplementary material for: Race, Gender, and Faculty Retention in Academic Medicine
Source: JAMA Netw Open. 2024 Nov 14;7(11):e2445143. doi: 10.1001/jamanetworkopen.2024.45143 (PMC11565262; doi:10.1001/jamanetworkopen.2024.45143)
Supplement: Supplement 1. — eFigure 1. Proportion Retained by Career Milestones eFigure 2. Survival Curve for Gender by Degree Type eFigure 3. Survival Curve for Degree Type eFigure 4. Survival Curve for Race and Ethnicity eFigure 5. Proportion Retained by Gender and Race/Ethnicity by Career Milestones eTable 1. Proportion Retained by Career Milestones eTable 2. Proportion of MD Only Faculty Retained by Career Milestones eTable 3. Proportion of PhD Only Faculty Retained by Career Milestones eTable 4. Proportion of Joint MD and PhD Faculty Retained by Career Milestones eTable 5. Proportion of Other Degree Faculty Retained by Career Milestones eTable 6. Bivariable Hazard Ratios for Attrition by Gender, Race, Degree Type, and Decade eTable 7. Proportion of Faculty Retained by Race and Ethnicity and Gender eTable 8. Cox PH Model Results With Interaction Between Gender and Degree Type, Including Race and Institutional Variables [file jamanetwopen-e2445143-s001.pdf]

Supplemental Online Content

Scheuermann TS, Clark L, Sultana N, et al. Race, gender, and faculty retention in academic medicine. *JAMA Netw Open*. 2024;7(11):e2445143. doi:10.1001/jamanetworkopen.2024.45143

eFigure 1. Proportion Retained by Career Milestones
eFigure 2. Survival Curve for Gender by Degree Type
eFigure 3. Survival Curve for Degree Type
eFigure 4. Survival Curve for Race and Ethnicity
eFigure 5. Proportion Retained by Gender and Race/Ethnicity by Career Milestones
eTable 1. Proportion Retained by Career Milestones
eTable 2. Proportion of MD Only Faculty Retained by Career Milestones
eTable 3. Proportion of PhD Only Faculty Retained by Career Milestones
eTable 4. Proportion of Joint MD and PhD Faculty Retained by Career Milestones
eTable 5. Proportion of Other Degree Faculty Retained by Career Milestones
eTable 6. Bivariable Hazard Ratios for Attrition by Gender, Race, Degree Type, and Decade
eTable 7. Proportion of Faculty Retained by Race and Ethnicity and Gender
eTable 8. Cox PH Model Results With Interaction Between Gender and Degree Type, Including Race and Institutional Variables

This supplemental material has been provided by the authors to give readers additional information about their work.

eFigure 1. Proportion Retained by Career Milestones

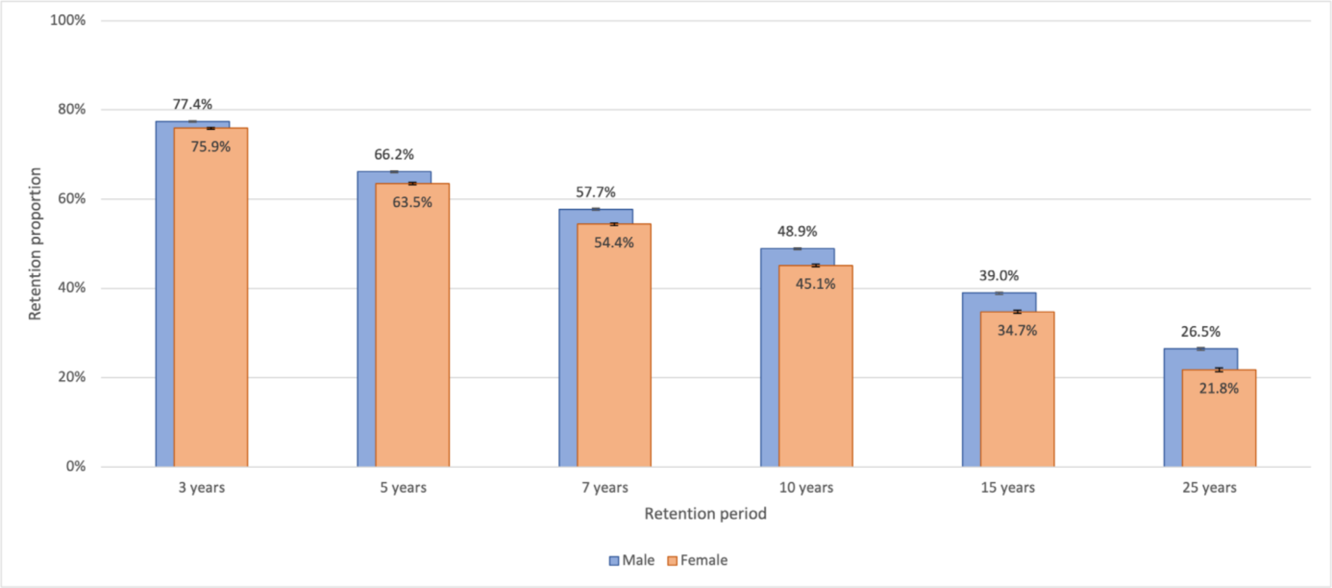

© 2024 Scheuermann TS et al. *JAMA Network Open.*

eFigure 2. Survival Curve for Gender by Degree Type

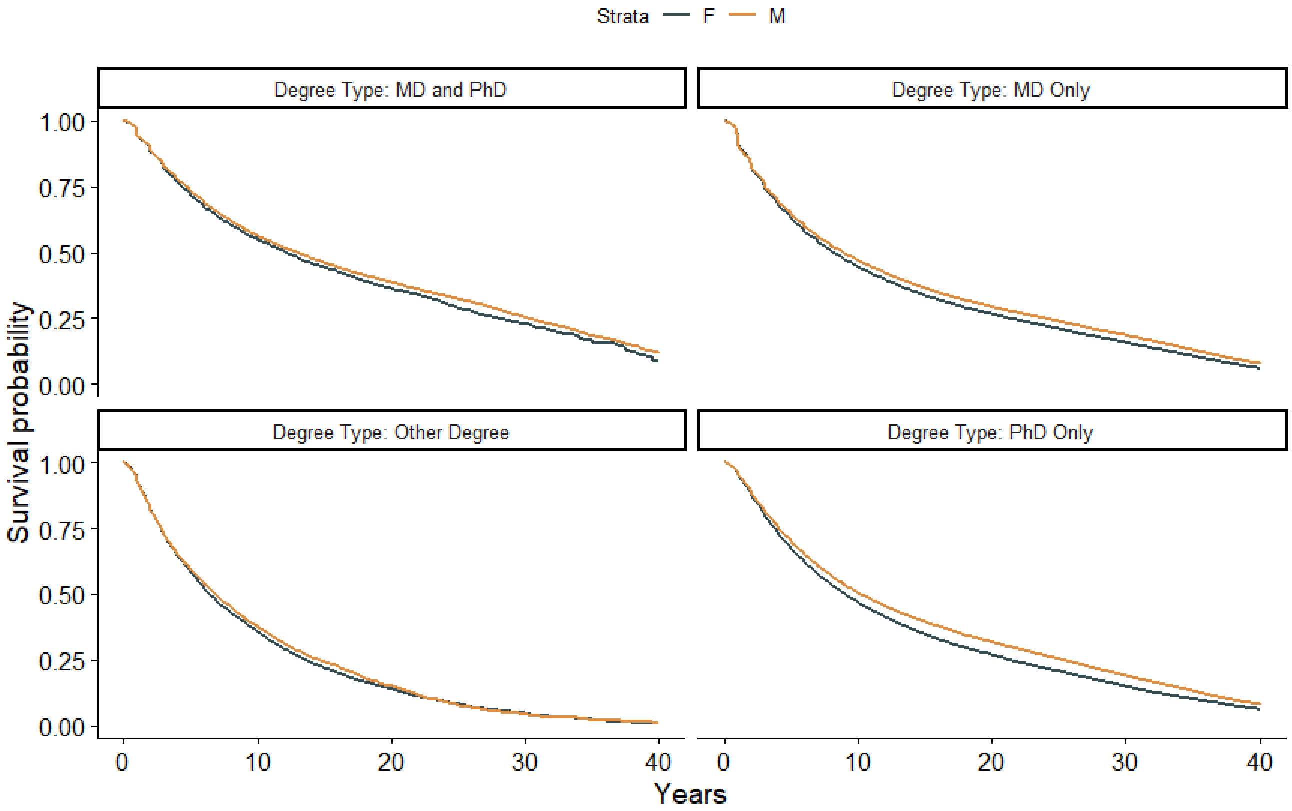

© 2024 Scheuermann TS et al. *JAMA Network Open.*

eFigure 3. Survival Curve for Degree Type

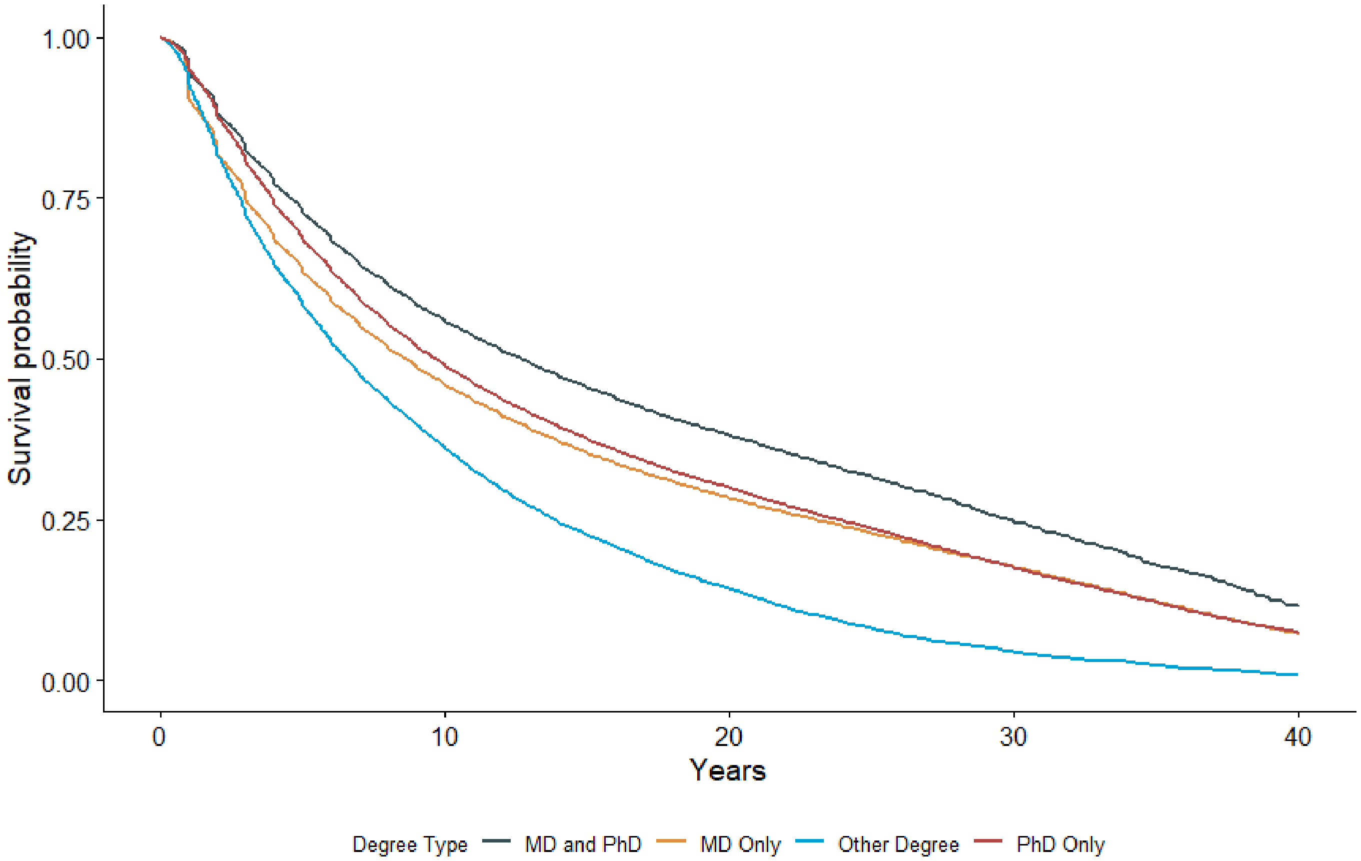

Notes. Median survival times for MD and PhD Degree Type: 12.67 (12.33, 13.00) years; MD Only Degree Type: 8.62 (8.50, 8.75) years; PhD Only Degree Type: 9.67 (9.50, 9.83) years; and Other Degree Type: 6.54 (6.37, 6.75) years.

© 2024 Scheuermann TS et al. *JAMA Network Open.*

eFigure 4. Survival Curve for Race and Ethnicity

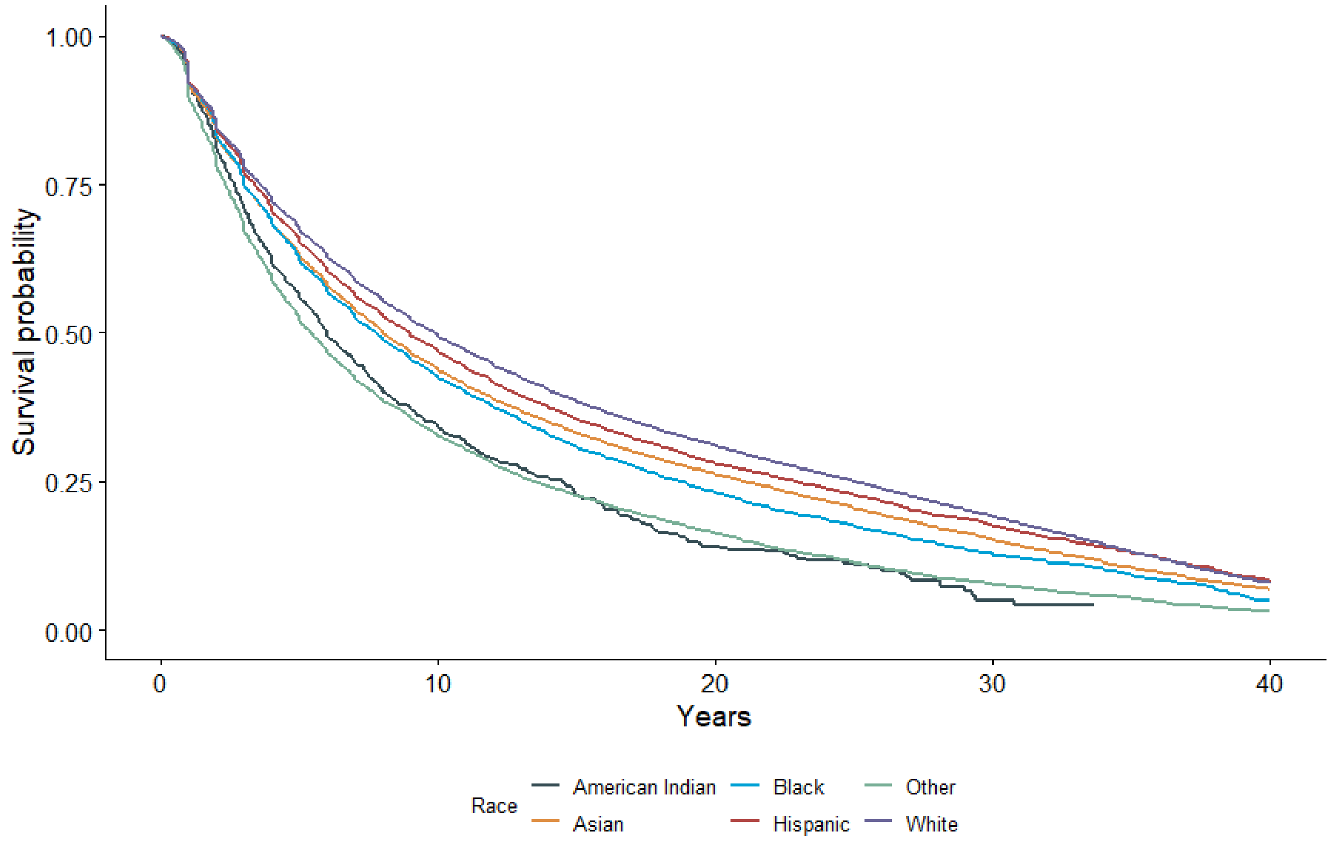

© 2024 Scheuermann TS et al. *JAMA Network Open.*

eFigure 5. Proportion Retained by Gender and Race/Ethnicity by Career Milestones

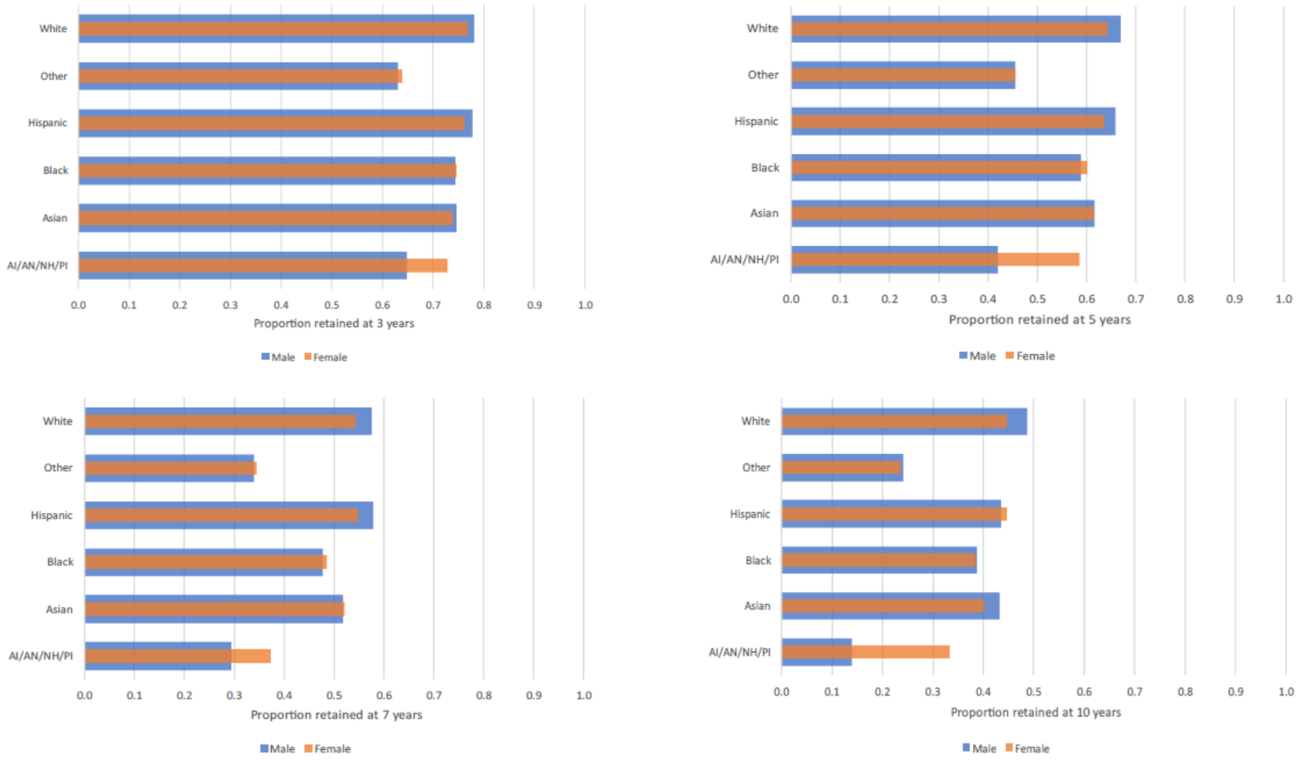

© 2024 Scheuermann TS et al. *JAMA Network Open.*

eTable 1. Proportion Retained by Career Milestones

	Women		Men	
	**Count Retained/At Risk of being Retained**	**Proportion Retained (95% Exact Binomial CI)**	**Count Retained/At Risk of being Retained**	**Proportion Retained (95% Exact Binomial CI)**
**Retained at least 3 Years**	107,494/141,580	0.7592 (0.7570, 0.7615)	167,716/216,627	0.7742 (0.7724, 0.7760)
**Retained at least 5 Years**	80,529/126,883	0.6347 (0.6320, 0.6373)	133,134/201,275	0.6615 (0.6594, 0.6635)
**Retained at least 7 Years**	61,428/112,919	0.5440 (0.5411, 0.5469)	78,695/186,195	0.5774 (0.5751, 0.5796)
**Retained at least 10 Years**	42,377/93,906	0.4513 (0.4481, 0.4545)	80,599/164,820	0.4890 (0.4866, 0.4914)
**Retained at least 15 Years**	23,220/66,865	0.3473 (0.3437, 0.3509)	51,163/131,280	0.3897 (0.3871, 0.3924)
**Retained at least 25 Years**	7,085/32,580	0.2175 (0.2130, 0.2220)	20,868/78,907	0.2645 (0.2614, 0.2676)

© 2024 Scheuermann TS et al. *JAMA Network Open.*

eTable 2. Proportion of MD Only Faculty Retained by Career Milestones

	Women		Men	
	**Count Retained/At Risk of being Retained**	**Proportion Retained (95% Exact Binomial CI)**	**Count Retained/At Risk of being Retained**	**Proportion Retained (95% Exact Binomial CI)**
**Retained at least 3 Years**	66,267/89,146	0.7434 (0.7405, 0.7462)	111,334/147,502	0.7548 (0.7526, 0.7570)
**Retained at least 5 Years**	49,333/79,248	0.6225 (0.6191, 0.6259)	87,470/136,368	0.6414 (0.6389, 0.6440)
**Retained at least 7 Years**	37,364/69,874	0.5347 (0.5310, 0.5384)	70,027/125,431	0.5583 (0.5555, 0.5610)
**Retained at least 10 Years**	25,480/57,182	0.4459 (0.4415, 0.4497)	52,011/110,236	0.4718 (0.4689, 0.4748)
**Retained at least 15 Years**	13,744/39,629	0.3468 (0.3421, 0.3515)	32,545/87,298	0.3728 (0.3696, 0.3760)
**Retained at least 25 Years**	4,332/19,273	0.2248 (0.2189, 0.2307)	13,561/53,628	0.2529 (0.2492, 0.2566)

© 2024 Scheuermann TS et al. *JAMA Network Open.*

eTable 3. Proportion of PhD Only Faculty Retained by Career Milestones

	Women		Men	
	**Count Retained/At Risk of being Retained**	**Proportion Retained (95% Exact Binomial CI)**	**Count Retained/At Risk of being Retained**	**Proportion Retained (95% Exact Binomial CI)**
**Retained at least 3 Years**	27,356/34,159	0.8008 (0.7966, 0.8051)	38,811/47,519	0.8167 (0.8132, 0.8202)
**Retained at least 5 Years**	20,824/31,121	0.6691 (0.6639, 0.6744)	31,263/44,608	0.7008 (0.6966, 0.7051)
**Retained at least 7 Years**	16,213/28,252	0.5739 (0.5681, 0.5796)	25,575/41,742	0.6127 (0.6080, 0.6174)
**Retained at least 10 Years**	11,556/24,341	0.4748 (0.4685, 0.4811)	19,405/37,488	0.5176 (0.5126, 0.5227)
**Retained at least 15 Years**	6,653/18,123	0.3671 (0.3601, 0.3742)	12,637/30,287	0.4172 (0.4117, 0.4228)
**Retained at least 25 Years**	2,074/8,911	0.2327 (0.2240, 0.2417)	5,120/17,953	0.2852 (0.2786, 0.2919)

© 2024 Scheuermann TS et al. *JAMA Network Open.*

eTable 4. Proportion of Joint MD and PhD Faculty Retained by Career Milestones

	Women		Men	
	**Count Retained/At Risk of being Retained**	**Proportion Retained (95% Exact Binomial CI)**	**Count Retained/At Risk of being Retained**	**Proportion Retained (95% Exact Binomial CI)**
**Retained at least 3 Years**	5,482/6,659	0.8232 (0.8139, 0.8323)	14,506/17,394	0.8340 (0.8284, 0.8395)
**Retained at least 5 Years**	4,373/6,061	0.7215 (0.7100, 0.7328)	12,146/16,474	0.7373 (0.7305, 0.7440)
**Retained at least 7 Years**	3,520/5,465	0.6441 (0.6312, 0.6568)	10,193/15,540	0.6559 (0.6484, 0.6634)
**Retained at least 10 Years**	2,594/4607	0.5631 (0.5486, 0.5774)	8,022/14,050	0.5710 (0.5627, 0.5792)
**Retained at least 15 Years**	1,508/3,236	0.4660 (0.4487, 0.4834)	5,357/11,259	0.4758 (0.4665, 0.4851)
**Retained at least 25 Years**	390/1,228	0.3176 (0.2916, 0.3444)	2,054/5,949	0.3453 (0.3332, 0.3575)

© 2024 Scheuermann TS et al. *JAMA Network Open.*

eTable 5. Proportion of Other Degree Faculty Retained by Career Milestones

	Women		Men	
	**Count Retained/At Risk of being Retained**	**Proportion Retained (95% Exact Binomial CI)**	**Count Retained/At Risk of being Retained**	**Proportion Retained (95% Exact Binomial CI)**
**Retained at least 3 Years**	8,389/11,616	0.7222 (0.7139, 0.7303)	3,065/4,212	0.7277 (0.7140, 0.7411)
**Retained at least 5 Years**	5,999/10,453	0.5739 (0.5644, 0.5834)	2,255/3,825	0.5895 (0.5738, 0.6052)
**Retained at least 7 Years**	4,331/9,328	0.4643 (0.4541, 0.4745)	1,705/3,482	0.4897 (0.4729, 0.5064)
**Retained at least 10 Years**	2,747/7,776	0.3533 (0.3426, 0.3640)	1,161/3,046	0.3812 (0.3639, 0.3987)
**Retained at least 15 Years**	1,315/5,877	0.2238 (0.2132, 0.2346)	624/2,436	0.2562 (0.2389, 0.2740)
**Retained at least 25 Years**	298/3,168	0.0912 (0.0814, 0.1018)	133/1,377	0.0966 (0.0815, 0.1134)

© 2024 Scheuermann TS et al. *JAMA Network Open.*

eTable 6. Bivariable Hazard Ratios for Attrition by Gender, Race, Degree Type, and Decade

Variable	Leaving Academia (Event)	Median Retention (95% CI)	Hazard Ratio (95% CI)
Gender			
Women (N=157,937)	92,904	8.33 (8.18, 8.41)	1.11 (1.10, 1.12)
Men (N=232,829)	147,377	9.35 (9.25, 9.42)	Reference
Degree Type			
MD Only (N=259,628)	158,740	8.62 (8.50, 8.75)	Reference
MD and PhD (N=25,453)	14,380	12.67 (12.33, 13.00)	0.75 (0.74, 0.76)
PhD Only (N=87,954)	55,004	9.67 (9.50, 9.83)	0.91 (0.91, 0.92)
Other Degree (N=17,731)	12,157	6.54 (6.36, 6.74)	1.36 (1.34, 1.39)
Race			
American Indian, Alaska Native, Native Hawaiian, or Pacific Islander (N=1,190)	822	6.00 (5.62, 6.62)	1.52 (1.42, 1.62)
Asian (N=72,490)	41,040	8.00 (8.00, 8.11)	1.15 (1.14, 1.17)
Black (14,920)	9,294	7.78 (7.50, 8.00)	1.21 (1.18, 1.23)
Hispanic, Latino, of Spanish Origin, or Multi-Race Hispanic (N=20,345)	11,432	9.00 (8.75, 9.16)	1.07 (1.05, 1.09)
White (N=251,670)	157,408	9.91 (9.83, 9.92)	Reference
Other, Multi-Race Non-Hispanic, or Unknown (N=30,151)	20,285	5.42 (5.33, 5.55)	1.58 (1.55, 1.60)
Decade			
70s (N=11,689)	5,977	9.50 (9.08, 10.00)	0.89 (0.87, 0.91)
80s (N=54,617)	28,563	9.16 (9.00, 9.33)	0.92 (0.91, 0.94)
90s (N=72,376)	36,922	9.75 (9.66, 9.92)	0.87 (0.86, 0.89)
00s (N=105,478)	56,285	9.00 (8.91, 9.00)	0.93 (0.92, 0.94)
10s (N=143,925)	54,548	8.00 (7.93, 8.00)	Reference

Note. Hazard ratios were generated from four separate unadjusted models. Follow-up periods for each unadjusted Cox Proportional Hazards Ratio Model for Gender, Degree Types, and Race include 40-year follow-up. The unadjusted model for decade includes only 10-year follow-up.

© 2024 Scheuermann TS et al. *JAMA Network Open.*

eTable 7. Proportion of Faculty Retained by Race and Ethnicity and Gender among Faculty with First Appointments in the 2010s

Race and Gender	**3-Year Retention**	**5-Year Retention**	**7-Year Retention**	**10-Year Retention**
	Proportion (95% CI)	Proportion (95% CI)	Proportion (95% CI)	Proportion (95% CI)
American Indian, Alaska Native, Native Hawaiian, or Pacific Islander Women	0.73 (0.66, 0.79)	0.59 (0.50, 0.67)	0.37 (0.28, 0.47)	0.33 (0.19, 0.51)
American Indian, Alaska Native, Native Hawaiian, or Pacific Islander Men	0.65 (0.58, 0.71)	0.42 (0.34, 0.50)	0.29 (0.21, 0.39)	0.14 (0.05, 0.30)
Asian Women	0.74 (0.73, 0.75)	0.61 (0.60, 0.62)	0.52 (0.51, 0.53)	0.40 (0.38, 0.43)
Asian Men	0.75 (0.74, 0.75)	0.62 (0.61, 0.62)	0.52 (0.51, 0.53)	0.43 (0.41, 0.46)
Black Women	0.75 (0.73, 0.76)	0.60 (0.58, 0.62)	0.48 (0.46, 0.51)	0.38 (0.33, 0.44)
Black Men	0.74 (0.72, 0.76)	0.59 (0.56, 0.62)	0.48 (0.44, 0.51)	0.39 (0.32, 0.45)
Hispanic Women	0.76 (0.75, 0.78)	0.64 (0.62, 0.65)	0.55 (0.52, 0.57)	0.45 (0.40, 0.50)
Hispanic, Latino, of Spanish Origin, or Multi-Race Hispanic Men	0.78 (0.76, 0.79)	0.66 (0.64, 0.68)	0.58 (0.56, 0.60)	0.43 (0.39, 0.48)
White Women	0.77 (0.76, 0.77)	0.64 (0.64, 0.65)	0.54 (0.53, 0.55)	0.45 (0.43, 0.46)
White Men	0.78 (0.78, 0.79)	0.67 (0.66, 0.67)	0.57 (0.57, 0.58)	0.49 (0.47, 0.50)
Other, Multi-Race Non-Hispanic, or Unknown Women	0.64 (0.63, 0.65)	0.45 (0.44, 0.47)	0.34 (0.33, 0.36)	0.24 (0.21, 0.27)
Other, Multi-Race Non-Hispanic, or Unknown Men	0.63 (0.62, 0.64)	0.45 (0.44, 0.47)	0.34 (0.32, 0.36)	0.24 (0.21, 0.27)

© 2024 Scheuermann TS et al. *JAMA Network Open.*

eTable 8. Cox PH Model Results With Interaction Between Gender and Degree Type, Including Race and Institutional Variables

Variable	Leaving Academia (Event)	Median Retention (95% CI)	Hazard Ratio (95% CI)
Gender			
MD Only			
Women (N=89,807)	52,801	7.00 (7.00, 1.09)	1.03 (1.02, 1.04)
Men (N=139,358)	90,506	7.29 (7.17, 7.41)	Reference
MD and PhD			
Women (N=6,034)	3,294	9.58 (9.00, 10.00)	1.00 (0.96, 1.04)
Men (N=14,526)	8,904	9.50 (9.16, 9.89)	Reference
PhD Only			
Women (N=34,471)	21,145	8.00 (7.92, 8.17)	1.07 (1.05, 1.09)
Men (N=44,593)	29,495	8.66 (8.49, 8.83)	Reference
Other Degree			
Women (N=12,976)	8,731	6.33 (6.17, 6.50)	1.03 (0.99, 1.08)
Men (N=4,519)	3,259	6.75 (6.42, 7.00)	Reference
Race			
American Indian, Alaska Native, Native Hawaiian, or Pacific Islander (N=1,097)	769	5.58 (5.00, 6.00)	1.44 (1.34, 1.54)
Asian (N=64,136)	37,525	6.88 (6.75, 6.92)	1.16 (1.15, 1.78)
Black (N=13,482)	8,565	6.92 (6.74, 7.00)	1.14 (1.12, 1.17)
Hispanic, Latino, of Spanish Origin, or Multi-Race Hispanic (N=18,142)	10,426	7.75 (7.42, 7.99)	1.09 (1.07, 1.11)
White (N=220,914)	141,339	8.33 (8.21, 8.41)	Reference
Other, Multi-Race Non-Hispanic, or Unknown (N=28,513)	19,511	5.00 (4.92, 5.00)	1.54 (1.52, 1.57)
Institution Ownership			
Private (N=169,443)	109,134	7.46 (7.39, 7.50)	1.09 (0.91, 1.30)
Public (N=176,841)	109,001	7.75 (7.66, 7.83)	Reference
Institution Revenue			
Private 1st Quartile (N=7,650)	4,986	8.34 (8.00, 8.70)	0.99 (0.78, 1.24)
Private 2nd Quartile (N=27,236)	18,784	7.83 (7.67, 7.96)	1.02 (0.84, 1.25)
Private 3rd Quartile (N=51,300)	32,434	7.07 (7.00, 7.17)	0.94 (0.77, 1.14)
Private 4th Quartile (N=82,757)	52,930	7.45 (7.33, 7.58)	
Public 1st Quartile (N=13,010)	6,956	8.78 (8.49, 9.00)	1.09 (0.89, 1.34)
Public 2nd Quartile (N=29,689)	21,209	6.75 (6.59, 6.91)	1.26 (1.06, 1.50)
Public 3rd Quartile (N=49,846)	30,885	7.27 (7.16, 7.42)	1.17 (1.00, 1.38)
Public 4th Quartile (N=84,296)	49,951	8.17 (8.04, 8.33)	Reference
Institution Size			
Small (N=44,612)	26,029	8.42 (8.25, 8.59)	0.81 (0.70, 0.94)
Medium (N=131,687)	85,725	7.00 (6.92, 7.00)	1.00 (0.89, 1.12)
Large (N=168,680)	106,309	7.92 (7.83, 7.92)	Reference

© 2024 Scheuermann TS et al. *JAMA Network Open.*

Variable	**Leaving Academia (Event)**	**Median Retention (95% CI)**	**Hazard Ratio (95% CI)**
Degree Type			
MD Only (N=227,648)	115,933	7.17 (7.08, 7.25)	Reference
MD and PhD (N=20,478)	9,426	9.50 (9.17, 9.83)	0.77 (0.75, 0.78)
PhD Only (N=78,257)	38,287	8.34 (8.25, 8.49)	0.80 (0.78, 1.03)
Other Degree (N=17,220)	9,480	6.42 (6.26, 6.59)	1.00 (0.98, 1.03)
Gender			
1970s			
Women (N=1,921)	1,219	5.92 (5.50, 6.17)	1.16 (1.08, 1.23)
Men (N=7,546)	4,335	7.00 (6.91, 7.42)	Reference
1980s			
Women (N=12,623)	7,679	6.59 (6.41, 6.83)	1.05 (1.02, 1.07)
Men (N=32,059)	18,809	7.00 (6.92, 7.17)	Reference
1990s			
Women (N=22,187)	12,741	7.92 (7.83, 8.08)	1.01 (0.99, 1.03)
Men (N=39,035)	22,032	8.08 (8.00, 8.25)	Reference
2000s			
Women (N=39,787)	23,643	7.16 (7.00, 7.29)	1.07 (1.05, 1.08)
Men (N=52,880)	29,894	7.95 (7.83, 8.00)	Reference
2010s			
Women (N=65,399)	25,373	7.28 (7.17, 7.43)	0.99 (0.98, 1.01)
Men (N=70,166)	27,401	7.62 (7.50, 7.75)	Reference
Race			
American Indian, Alaska Native, Native Hawaiian, or Pacific Islander (N=1,090)	663	5.58 (5.00, 6.00)	1.43 (1.33, 1.55)
Asian (N=63,486)	32,269	6.89 (6.75, 6.92)	1.17 (1.16, 1.19)
Black (N=13,366)	6,964	6.92 (6.74, 7.00)	1.13 (1.11, 1.16)
Hispanic, Latino, of Spanish Origin, or Multi-Race Hispanic (N=17,965)	8,654	7.75 (7.42, 7.95)	1.09 (1.07, 1.12)
White (N=219,509)	107,633	8.31 (8.18, 8.41)	Reference
Other, Multi-Race Non-Hispanic, or Unknown (N=28,187)	16,943	5.00 (4.92, 5.00)	1.60 (1.57, 1.62)
**Institution Ownership**			
Private (N=168,228)	86,364	7.43 (7.36, 7.50)	1.03 (0.84, 1.27)
Public (N=175,375)	86,762	7.75 (7.66, 7.83)	Reference
**Institution Revenue**			
Private 1st Quartile (N=7,576)	3,682	8.34 (8.00, 8.68)	0.98 (0.76, 1.26)
Private 2nd Quartile (N=27,482)	14,046	7.83 (7.67, 7.92)	1.02 (0.82, 1.28)
Private 3rd Quartile (N=50,931)	26,287	7.05 (7.00, 7.17)	1.00 (0.80, 1.24)
Private 4th Quartile (N=82,239)	42,349	7.42 (7.33, 7.56)	1.04 (1.04, 1.04)
Public 1st Quartile (N=12,901)	5,555	8.78 (8.46, 9.00)	1.06 (0.85, 1.34)
Public 2nd Quartile (N=29,498)	16,470	6.75 (6.59, 6.91)	1.23 (1.01, 1.49)
Public 3rd Quartile (N=49,362)	24,840	7.25 (7.16, 7.42)	1.16 (0.97, 1.40)
Public 4th Quartile (N=83,614)	39,897	8.17 (8.01, 8.33)	Reference
**Institution Size**			
Small (N=44,258)	20,098	8.42 (8.25, 8.59)	0.81 (0.69, 0.95)
Medium (N=130,828)	69,174	7.00 (6.92, 7.00)	1.02 (0.90, 1.16)
Large (N=167,361)	83,783	7.92 (7.83, 7.92(	Reference
