# Supplementary material for: Race, Gender, and Faculty Retention in Academic Medicine
Source: JAMA Netw Open. 2024 Nov 14;7(11):e2445143. doi: 10.1001/jamanetworkopen.2024.45143 (PMC11565262; doi:10.1001/jamanetworkopen.2024.45143)
Supplement: Supplement 2. — Data Sharing Statement [file jamanetwopen-e2445143-s002.pdf]

Data Sharing Statement

Scheuermann. Race, Gender and Faculty Retention in Academic Medicine. *JAMA Netw Open*. Published November 14, 2024. doi:10.1001/jamanetworkopen.2024.45143

Data
Data available: No

Additional Information
Explanation for why data not available: Data is available from the Association of American Medical Colleges ("AAMC") through their data sharing request process.
